# Insect Cell-Expressed Major Ragweed Allergen Amb a 1.01 Exhibits Similar Allergenic Properties to Its Natural Counterpart from Common Ragweed Pollen

**DOI:** 10.3390/ijms25105175

**Published:** 2024-05-09

**Authors:** Maria-Roxana Buzan, Manuela Grijincu, Lauriana-Eunice Zbîrcea, Laura Haidar, Tudor-Paul Tamaș, Monica-Daniela Cotarcă, Gabriela Tănasie, Milena Weber, Elijahu Babaev, Frank Stolz, Rudolf Valenta, Virgil Păunescu, Carmen Panaitescu, Kuan-Wei Chen

**Affiliations:** 1 Center of Immuno-Physiology and Biotechnologies, Department of Functional Sciences, Victor Babes University of Medicine and Pharmacy, 300041 Timisoara, Romania; buzan.roxana@umft.ro (M.-R.B.); grijincu.manuela@umft.ro (M.G.); zbircea.lauriana@umft.ro (L.-E.Z.);; 2 OncoGen Center, Pius Brinzeu County Clinical Emergency Hospital, 300723 Timisoara, Romania; kuan-wei.chen@oncogen.ro; 3 Division of Immunopathology, Department of Pathophysiology and Allergy Research, Center for Pathophysiology, Infectiology and Immunology, Medical University of Vienna, 1090 Vienna, Austria; 4 Vienna Competence Center, Biomay AG, 1090 Vienna, Austria; 5 Laboratory for Immunopathology, Department of Clinical Immunology and Allergology, Sechenov First Moscow State Medical University, 119991 Moscow, Russia; 6 Karl Landsteiner University of Health Sciences, 3500 Krems, Austria; 7 NRC Institute of Immunology FMBA of Russia, 115478 Moscow, Russia

**Keywords:** allergy, allergen, recombinant allergen, ragweed pollen allergy, Amb a 1, molecular diagnosis, specific IgE

## Abstract

Common ragweed pollen allergy has become a health burden worldwide. One of the major allergens in ragweed allergy is Amb a 1, which is responsible for over 90% of the IgE response in ragweed-allergic patients. The major allergen isoform Amb a 1.01 is the most allergenic isoform in ragweed pollen. So far, no recombinant Amb a 1.01 with similar allergenic properties to its natural counterpart (nAmb a 1.01) has been produced. Hence, this study aimed to produce a recombinant Amb a 1.01 with similar properties to the natural isoform for improved ragweed allergy management. Amb a 1.01 was expressed in insect cells using a codon-optimized DNA construct with a removable *N*-terminal His-Tag (rAmb a 1.01). The recombinant protein was purified by affinity chromatography and physicochemically characterized. The rAmb a 1.01 was compared to nAmb a 1.01 in terms of the IgE binding (enzyme-linked immunosorbent assay (ELISA), immunoblot) and allergenic activity (mediator release assay) in well-characterized ragweed-allergic patients. The rAmb a 1.01 exhibited similar IgE reactivity to nAmb a 1.01 in different IgE-binding assays (i.e., IgE immunoblot, ELISA, quantitative ImmunoCAP inhibition measurements). Furthermore, the rAmb a 1.01 showed comparable dose-dependent allergenic activity to nAmb a 1.01 regarding basophil activation. Overall, the results showed the successful expression of an rAmb a 1.01 with comparable characteristics to the corresponding natural isoform. Our findings provide the basis for an improvement in ragweed allergy research, diagnosis, and immunotherapy.

## 1. Introduction

Short ragweed (*Ambrosia artemisiifolia)* is an invasive plant native to North America that has spread worldwide [1,2,3,4,5,6,7,8,9,10,11,12,13]. Due to its high allergenicity, ragweed pollen has become a significant health issue in infested areas, causing severe respiratory symptoms [14]. Ragweed-allergic patients exhibit heterogeneous and complex sensitization patterns [15] involving at least 11 described allergens [2].

Among the 11 ragweed allergenic proteins described in the WHO/IUIS allergen nomenclature database [16], Amb a 1 is the major allergen, inducing IgE sensitization in more than 90% of ragweed pollen-sensitized individuals [2,17]. Moreover, Amb a 1-specific IgE accounts for more than 50% of ragweed pollen-specific IgE levels in the majority of ragweed pollen-allergic patients [18]. It is also a very abundant protein, comprising up to 15% of the total protein content within ragweed pollen [19] and between 54 and 78% of its allergenic content [20,21]. 

Amb a 1, formerly known as antigen E, is an acidic glycoprotein [22] with a molecular weight of approximately 38 kDa [23,24]. During the purification process, the natural Amb a 1 allergen can undergo proteolytic cleavage, resulting in two units, a 26 kDa *C*-terminal alpha chain (amino acids 181–396) and a 12 kDa *N*-terminal beta chain (amino acids 26–181) [23,25]. Compared to the natural whole allergen, the alpha chain displays decreased IgE reactivity but comparable T-cell reactivity, while the beta chain demonstrates similar IgE reactivity but reduced T-cell reactivity [26].

Currently, five Amb a 1 isoforms are described and registered in the WHO/IUIS allergen nomenclature database [27], with sequence identities between 63 and 86% [2]. This includes the former Amb a 2 allergen, which was reclassified as Amb a 1.05 based on the high sequence similarity with other Amb a 1 isoforms [28,29]. The immunological characterization of these five isoforms showed varying capacities for IgE binding [30], indicating that isoform Amb a 1.01 exhibits the highest allergenic activity and Amb a 1.01 and Amb a 1.03 are the most potent T cell stimulators [21,31,32].

At present, in vitro diagnosis and allergen-specific immunotherapy are mainly based on allergenic extracts. Although the quality of these extracts can be analyzed to some extent [33], their natural origin results in significant heterogeneity, leading to differences in both the composition and the quantity of allergenic proteins [34]. Also, the extracts may be contaminated with allergens from other sources [35]. The shortcomings of allergenic extracts can be overcome by using recombinant allergens with similar structures, functions, and immunological properties to their natural counterparts [36]. The use of defined recombinant allergens resembling the allergenic features would greatly improve allergy diagnosis. Indeed, in vitro molecular allergy diagnosis has been implemented worldwide, but there is an unmet need for in vivo diagnostics based on recombinant allergens [36,37,38]. Furthermore, the development of advanced forms of molecular allergen-specific immunotherapy (AIT) needs to progress more quickly [39,40].

Obtaining defined and functional Amb a 1 isoforms to serve as suitable recombinant allergens has proven to be a challenge and, so far, has been not completely successful. Attempts to express Amb a 1 isoforms in *Escherichia coli* cells resulted in improperly folded proteins with reduced IgE reactivity [26,41,42]. So far, only recombinant Amb a 1.03 obtained in *Pichia pastoris* has an IgE-binding capacity comparable to the native form [21]. However, no recombinant Amb a 1.01, which represents the most allergenic protein in ragweed pollen, is available. Therefore, the aim of our study was to produce Amb a 1.01 as a recombinant protein that behaves similarly to the natural allergen. For this purpose, Amb a 1.01 was expressed in insect cells, purified by affinity chromatography, characterized biochemically and immunologically, and compared regarding its allergenic properties to the natural isoform. Our study is the first to report a recombinant Amb a 1.01 isoform corresponding to the natural allergen and hence provides a basis for the advancement of molecular allergy diagnosis and AIT for ragweed pollen allergy. 

## 2. Results

### 2.1. Characterization of Recombinant Amb a 1.01 

The mature form of Amb a 1.01 was recombinantly produced as a soluble protein in *Spodoptera frugiperda (Sf9)* insect cells. The recombinant protein featured an *N*-terminal His-Tag, followed by an amino acid sequence (Glu-Asn-Leu-Tyr-Phe-Gln-Gly) designed for the His-Tag cleavage. After the His-Tag removal, an amino acid (glycine) remained at the *N*-terminal end of the recombinant allergen. The yield of the rAmb a 1.01 production was 1 mg of secreted protein per 400 mL of insect cell culture. 

The identity, purity, and isoform composition of the nAmb a 1.01 was confirmed by mass spectrometry and it was found to consist of 94% isoform 01, 4% isoform 04, and 1% isoform 02 [21].

**Figure 1:**
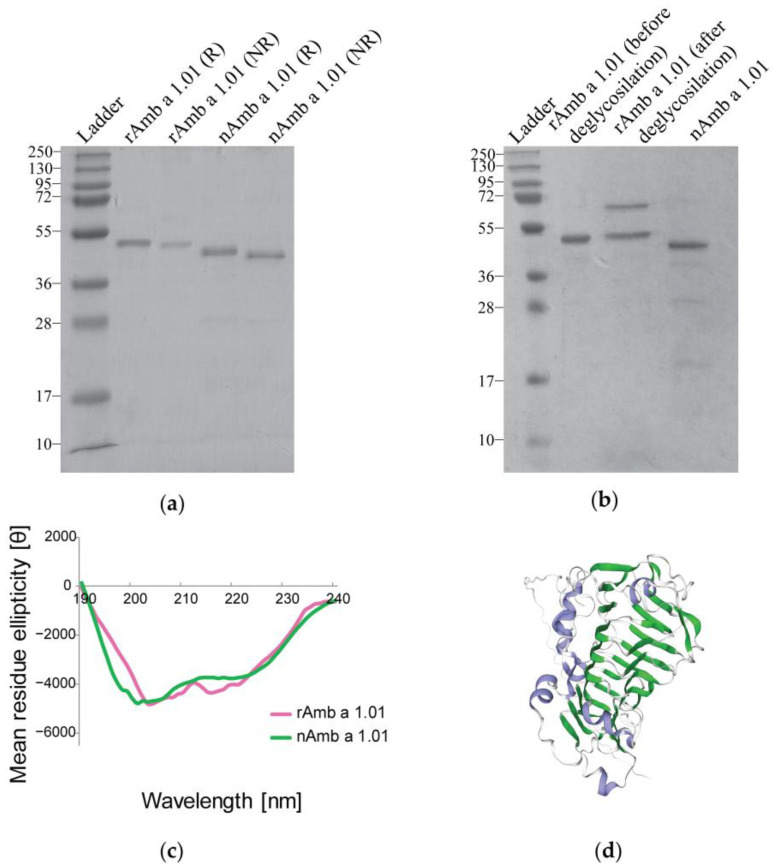
Comparison of the rAmb a 1.01 allergen with nAmb a 1.01. (**a**) Coomassie-stained SDS-PAGE under reducing (R) and non-reducing conditions (NR) with nAmb a 1.01 and rAmb 1.01. (**b**) SDS-PAGE comparison between rAmb a 1.01 before and after deglycosylation with PNGase A and nAmb a 1.01. Molecular weight (kDa) markers are indicated on the left. (**c**) The far UV spectrum of rAmb a 1.01 (pink), and the reference nAmb a 1.01 (green). The mean residual ellipticity θ (deg x cm^2^/dmol) is represented on the y-axis and the wavelength in nanometers (nm) on the x-axis. (**d**) Three-dimensional structural model of Amb a 1.01 from https://swissmodel.expasy.org/ (accessed on 15 January 2024; accession number P27759) [43], blue—alpha helices, green—beta strands, gray—turns and loops.

The sodium dodecyl sulfate-polyacrylamide gel electrophoresis (SDS-PAGE) comparative analysis of the recombinant protein and natural Amb a 1.01, under both reducing (R) and non-reducing (NR) conditions, showed a strong band at around 40 kDa for nAmb a 1.01, while the recombinant allergen displayed a strong band slightly above the band observed for the natural isoform (Figure 1a). This band corresponded well with the molecular weight predicted for Amb a 1.01 (i.e., 39.84 kDa), which showed a varying degree of sequence identity (63.33–86.9%) with the other known Amb a 1 isoforms (i.e., Amb a 1.02, Amb a 1.03, Amb a 1.04 and Amb a 1.05) (Figure S1).

The matrix-assisted laser desorption/ionization-time of flight (MALDI-TOF) analysis displayed a molecular weight of 41,040.87 Da for the rAmb a 1.01 and a molecular weight of 40,975.02 Da for the nAmb a 1.01 (Figure S2).

After the treatment with PNGase A, no changes in the molecular weight were observed between the recombinant protein before and after deglycosylation. The band at around 70 kDa corresponded to the PNGase A (Figure 1b). 

The circular dichroism (CD) measurements for the far UV spectrum showed that nAmb a 1.01 and rAmb a 1.01 had curves with comparable steepness, with a minimum between 201 and 211 nm and a maximum at 190 nm for rAmb a 1.01, and a minimum between 198 and 209 nm and a maximum at 190 nm for nAmb a 1.01 (Figure 1c). 

The DichroWeb CDSSTR method calculations using the CD spectra data indicated that rAmb a 1.01 consisted of 5% α-helix, 29% β-strands, 18% turns and 47% random coils. The natural Amb a 1.01 used as a reference consisted of 4% α-helix, 29% β-strands, 18% turns and 46% random coils. The 3D structure model generated in the Swiss model also showed a high content of β-strand structures in Amb a 1 (Figure 1d).

### 2.2. IgE Reactivity of the Recombinant Amb a 1.01 Allergen

**Figure 2:**
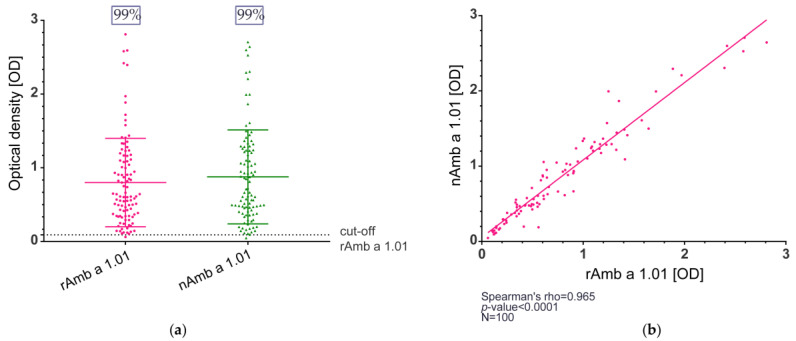
IgE reactivity of natural and recombinant Amb a 1.01 allergens determined by ELISA. (**a**) IgE binding frequency of rAmb a 1.01 and nAmb a 1.01. The cut-off was calculated for each allergen as the mean value + 3 × SD of the 5 non-allergic controls. The plot depicts the optical density (OD) measured in the ELISA (y-axis), the main horizontal line represents the median, and the other two lines mark the interquartile range (**b**) Correlation of the OD values for rAmb a 1.01 (x-axis) and nAmb a 1.01 (y-axis).

The IgE reactivity of natural Amb a 1.01 and recombinant Amb a 1.01 was determined in enzyme-linked immunosorbent assay (ELISA) and immunoblot experiments. The study population comprised 100 ragweed-allergic patients, of whom 37% were monosensitized and 63% polysensitized, and the other sensitizations are shown in Table S1. In the ELISA, the IgE binding for rAmb a 1.01 ranged between the optical density (OD) values of 0.061 and 2.811 (median OD = 0.611), and the OD values for nAmb a 1.01 were between 0.047 and 2.706 (median OD = 0.688) (Figure 2a, Table S1).

The IgE recognition frequency in the study population of ragweed-allergic patients was 99% for both the natural and the recombinant Amb a 1.01 (Figure 2a, Table S1). Furthermore, when comparing the OD values between the natural and the recombinant Amb a 1.01, a strong correlation was observed (Spearman’s rho = 0.965, *p*-value < 0.0001) (Figure 2b).

**Figure 3:**
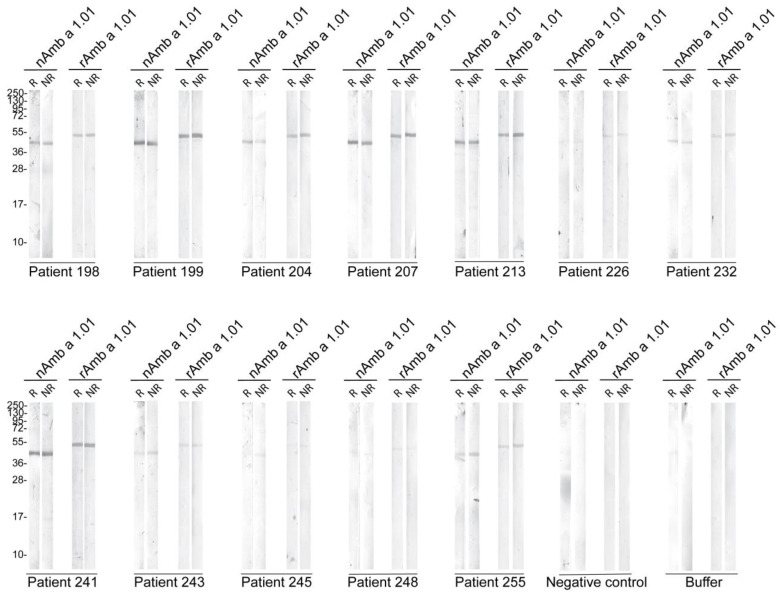
Immunoblot assay for 12 ragweed allergic patients toward nAmb a 1.01 and rAmb a 1.01 under reducing (R) and non-reducing conditions (NR). Serum from a non-allergic patient and buffer control were used as negative controls. Molecular weight (kDa) markers are indicated on the left.

Based on the ELISA results, sera from 12 Amb a 1.01 allergic patients were selected and their IgE reactivity was tested toward blotted reduced (R) and non-reduced (NR) nAmb a 1.01 and rAmb a 1.01. Serum from a non-allergic individual and buffer were included as negative controls. For nAmb a 1.01, all the patients reacted with different intensities, with a band at around 40 kDa. The patients’ sera IgE reacted with similar intensities to rAmb a 1.01, but the corresponding band migrated at a slightly higher molecular weight than nAmb a 1.01. No difference was observed in the IgE binding of the reduced and non-reduced nAmb a 1.01 and rAmb a 1.01. No IgE binding was observed for the buffer and negative control serum (Figure 3).

### 2.3. Allergenic Activity of Recombinant Amb a 1.01 

**Figure 4:**
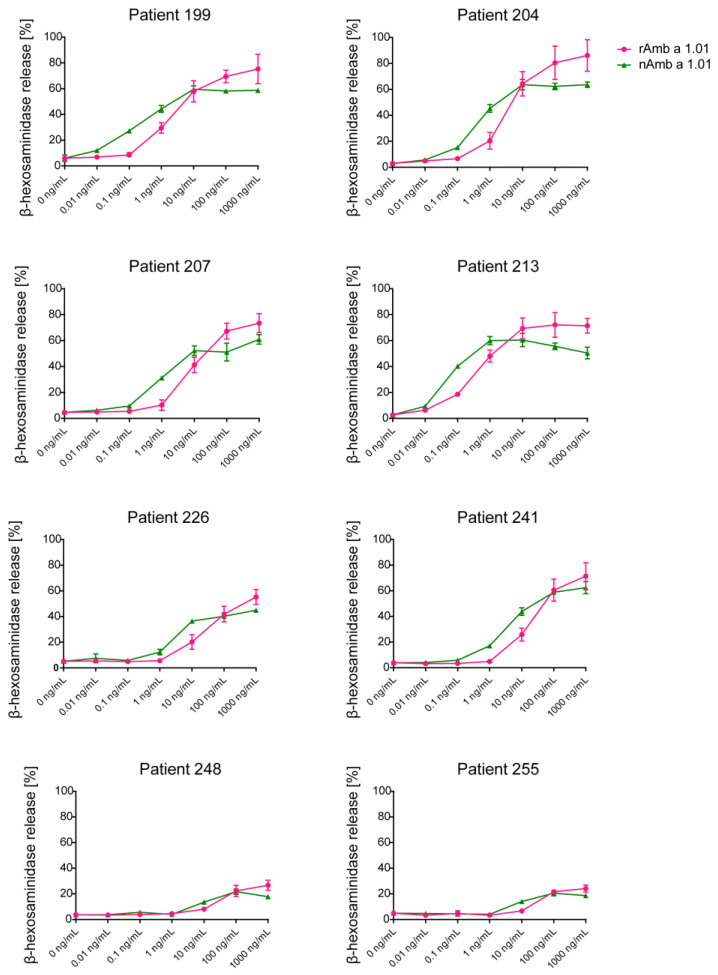
Assessment of the allergenic activity of natural and recombinant Amb a 1.01. The mediator release from RBL cells was triggered by serial dilutions (0.01 ng/mL–1000 ng/mL) of nAmb a 1.01 and rAmb a 1.01 (x-axes). The β-hexosaminidase releases are expressed as percentages of the total mediator contents +/− SD (y-axes).

The allergenic activity of rAmb a 1.01 was evaluated in comparison to nAmb a 1.01 using rat basophil leukemia (RBL) cells expressing human FcεRI and sera from eight Amb a 1.01 reactive patients (199, 204, 207, 213, 226, 241, 248, 255) (Figure 4). 

Natural Amb a 1.01 was able to induce dose-dependent mediator release in all the tested patients with different intensities (Figure 4, Table S2). Of note, in all the tested patients, the mediator release in the increasing part of the bell-shaped degranulation curve was detected to compare the allergenic activity of the natural and recombinant Amb a 1.01. The lowest concentration of nAmb a 1.01 that induced degranulation was 0.1 ng/mL when RBL cells were loaded with sera from patients with high Amb a 1-specific IgE levels in the ELISA (e.g., patients 213 and 199). The dose–response curves of the mediator release were comparable between nAmb a 1.01 and rAmb a 1.01 in all the tested patients. Interestingly, at high allergen concentrations, a higher degranulation percentage was observed with rAmb a 1.01 whereas at lower concentrations nAmb a 1 seemed to induce higher degranulation (Figure 4, Table S2). Patients with low Amb a 1-specific IgE levels as determined by ELISA and quantitative ImmunoCAP measurements (Table S3) (e.g., patients 248 and 255) showed low basophil activation.

### 2.4. IgE Inhibition with rAmb a 1.01 and Ragweed Pollen Extract in ImmunoCAP

**Figure 5:**
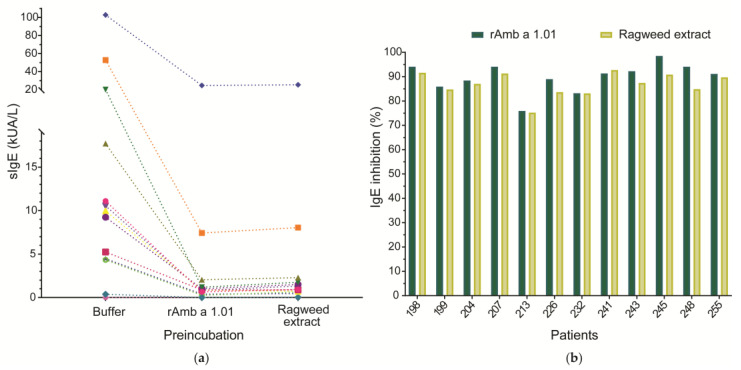
Inhibition of 12 allergic patients’ IgE binding to a commercially available nAmb a 1 preparation incubation with rAmb a 1.01 or ragweed pollen extract. (**a**) Amb a 1-specific IgE levels (y-axis) after pre-incubation of sera with rAmb a 1.01, ragweed pollen extract and DPBS (buffer) (x-axis). Each symbol represents a patient and the values are displayed in Table S3. The dotted line is used to connect the specific IgE values of the same patient after pre-incubation with different antigens. (**b**) The percentage of IgE inhibition is displayed on the y-axis for each patient (x-axis).

The percentage of Amb a 1.01 isoform-specific IgE directed to the natural Amb a 1 preparation in commercially available Amb a 1 tests was determined by an ImmunoCAP inhibition assay using sera from 12 Amb a 1 allergic patients (198, 199, 204, 207, 213, 226, 232, 241, 243, 245, 248, 255) and rAmb a 1.01 (100 µg/mL) as an inhibitor. For comparison, a pre-incubation of patients’ sera with ragweed pollen extract (1000 µg/mL) was also performed. Serum from a non-allergic (NC) individual was used as a negative control (Figure 5a, Table S3). 

The ImmunoCAP inhibition experiment using rAmb a 1.01 as an inhibitor showed an IgE inhibition of the nAmb a 1 preparation between 75.92 and 98.43% (mean inhibition 89.82%). When the sera were pre-incubated with ragweed extract, an inhibition of IgE binding to the nAmb a 1 preparation ranging between 75.23 and 92.71% (mean inhibition 86.86%) was obtained (Figure 5b, Table S3). The lowest inhibition (75.92%) was observed for patient 213, the one with the highest IgE level (>100 kUA/L), while the highest inhibition (98.43%) was observed for patient 245, the one with the lowest IgE level (0.383 kUA/L). For most of the patients, IgE binding to the nAmb a 1 preparation was inhibited better with rAmb a 1.01 than with ragweed pollen extract. Only patient 241 had a slightly higher inhibition after incubation with pollen extract (92.71%) than with rAmb a 1.01 (91.32%) (Figure 5b, Table S3).

The non-allergic (NC) subject displayed no Amb a 1-specific IgE (Table S3).

## 3. Discussion

Ragweed pollen allergy has become a major health problem worldwide and allergic patients display a complex and heterogeneous sensitization profile involving several allergenic molecules [15]. Due to the shortcomings of allergenic extracts [34] an accurate characterization of these profiles requires the use of pure allergen molecules with defined allergenic properties. Ideally, such allergens are available in the form of recombinant allergens that can be produced in high quantities, with reproducible quality and resembling the properties of the corresponding natural allergens. Such allergen molecules are important tools for improving ragweed allergy management, and in particular, for diagnosis. Furthermore, they can serve as benchmark molecules for the development of innovative molecular allergy vaccines [36,37,38,44]. Currently, 11 allergens have been described for ragweed pollen [16] and Amb a 1 is considered the major allergen, with more than 90% of ragweed allergic patients sensitized to it [2,17,45]. Amb a 1 has five known isoforms [27] with different IgE-binding capacities [30], among which Amb a 1.01 reportedly has the highest allergenic activity, indicating that it is the most important component in diagnostic tests and a major benchmark for AIT developments [21]. However, until now, this isoform has not been produced as a recombinant allergen equaling the allergenic properties of the natural allergen. Amb a 1.01 has only been isolated from ragweed pollen extract [21]. The purified natural allergens can present some disadvantages, such as the large amount of starting material that has to be collected and may be contaminated with other materials [36,46]. Furthermore, nAmb a 1.01 preparations are never completely pure and can contain other isoforms [21,47]. Our study is the first to report the recombinant expression of Amb a 1.01 with similar immunological characteristics to the natural allergen.

The baculovirus–insect cell expression system was chosen because previous studies using the *E. coli* expression system did not obtain a fully functional Amb a 1 allergen [26,41,42,48]. The insect cell expression system is widely used for the production of proteins containing disulfide bonds and post-translational modifications [36,49,50,51,52,53]. Peptide tags attached to proteins only rarely induce modifications in the secondary structure or biological functionality of the recombinant protein [54,55,56]. In order to exclude the potential interference of the His-Tag in the protein structure and to obtain a protein similar in sequence to the natural isoform, the His-Tag from rAmb a 1.01 was removed by protease cleavage after the protein purification. 

The SDS-PAGE analysis revealed that nAmb a 1.01 migrated at around 40 kDa, close to its theoretical molecular weight of 39.84 kDa (calculated with Expasy ProtParam [57]), while the recombinant allergen migrated slightly higher than the natural isoform even though its theoretical molecular weight is just 39.90 kDa (calculated with Expasy ProtParam [57]) (Figure 1a). This molecular weight difference can be explained by the presence of the amino acid glycine at the *N*-terminus of the rAmb a 1.01 sequence. Glycine is a hydrophobic amino acid and may affect the migration behavior of the protein in SDS-PAGE, but the determinations of the molecular weights of the recombinant and natural Amb a 1.01 by mass spectrometry demonstrate only a minor difference regarding one amino acid. The fact that both the recombinant and natural Amb a 1.01 had a slightly higher molecular weight when measured by mass spectrometry as compared to the predicted molecular weight may be explained by glycosylation. Although evidence was provided that natural Amb a 1 is not glycosylated [58], the amino acid sequence of Amb a 1.01 has an *N*-linked glycosylation site at position 36 [22]. Even though an attempt at deglycosylation with PNGase A did not change the molecular weight observed by SDS-PAGE, a possible glycosylation cannot be ruled out (Figure 1b).

The far UV CD spectroscopy results showed that the rAmb a 1.01’s CD spectrum was similar to the one for nAmb a 1.01 and exhibited the characteristic shape of proteins featuring a significant beta-sheet content (29%), which corresponds to the model of the three-dimensional structure of Amb a 1. The high amount of beta-sheets specific to Amb a 1.01 isoform was also reported by other studies, confirming that the predominant structural element within family 1 of polysaccharide lyases is a central parallel beta-helix [21,59] (Figure 1d). 

In terms of IgE binding, the natural Amb a 1.01 exhibited a high IgE-binding frequency (99%). Comparable reactivity percentages were reported in previous studies [17,21]. In comparison to nAmb a 1.01, rAmb a 1.01 displayed the same IgE frequency (99%) and the IgE levels specific to rAmb a 1.01 and nAmb a 1.01 were highly correlated (Figure 2b). Although the immunoblot results showed that the IgE reactivity and the intensities of the IgE recognition of the recombinant and natural bands were comparable in each tested patient, no correlation was observed when comparing the results of some patients via immunoblot and ELISA/RBL assay. For example, patients 248 and 255 had similar results in the ELISA and RBL assays, but patient 248 had a lower IgE binding in the immunoblot. A potential explanation is that the SDS-PAGE denaturing conditions might disrupt the allergen structure and thus hinder the recognition of the conformational epitopes and the IgE binding [60]. 

The most crucial test for evaluating the allergenic activity of a protein is the basophil activation test because it allows assessment of the ability of an allergen to induce IgE-mediated release of mediators. This test is very sensitive and can be used to study the dose dependency of the allergenic response. We found that the recombinant and natural protein induced comparable basophil activation curves, indicating that they are similar in terms of allergenic activity (Figure 4). Only in a few patients did the natural allergen seem to be a bit more potent in activating basophils at low concentrations. The recombinant protein provided different sigmoidal curves when tested with different patients. This can be attributed to the different allergen-specific IgE levels in the patients’ sera, the different number of IgE epitopes recognized by the sera, the different orientations of the IgE epitopes and/or the avidities of the IgE antibodies [61,62,63].

Regarding the potential cross-reactivity of Amb a 1 with other pectate lyases, there is only one relevant cross-reactive allergen, which is Art v 6 from mugwort pollen [64], but only 20% of the patients within the study population were sensitized to mugwort pollen (Table S1).

Overall, the IgE-binding assessment (ELISA, immunoblot, RBL cells’ mediator release) revealed that rAmb a 1.01 exhibits similar IgE reactivity and allergenic activity as compared to nAmb a 1.01. Results from the quantitative IgE inhibition experiments performed by IgE ImmunoCAP measurements indicated that rAmb a 1.01 accounted for the majority of IgE epitopes present in a nAmb a 1 preparation containing different isoforms. Only in a few patients was the inhibition of IgE binding to the nAmb a 1 preparation by rAmb a 1.01 incomplete, demonstrating that it is the most potent Amb a 1 isoform. 

The ImmunoCAP inhibition assay thus revealed the high IgE-binding inhibition potential of rAmb a 1.01 (Figure 5), suggesting that either the commercially available nAmb a 1 ImmunoCAP contains large amounts of Amb a 1.01 or the patients are mainly sensitized to this isoform. Either way, rAmb a 1.01 seems to be a suitable candidate for ragweed pollen allergy diagnosis, but more detailed studies including other isoforms of the major allergen Amb a 1 will be of interest.

## 4. Materials and Methods

### 4.1. Patients’ Sera 

Sera from 100 ragweed-allergic patients (#156–255) and 5 non-allergic individuals were obtained from an allergy center in Timisoara, Romania. The ragweed-allergic patients were characterized by a case history indicative of seasonal ragweed allergy, positive skin prick test and/or serum tests for ragweed-specific IgE. Written informed consent was obtained from each individual and the usage of sera for this study was approved by the Local Ethics Commission of Scientific Research of the Pius Brinzeu Emergency County Hospital Timisoara (ethical approval number 102, 10.01.2017). All the experiments were performed following relevant guidelines and regulations.

### 4.2. Recombinant Amb a 1.01 Allergen Expression

A codon-optimized DNA construct encoding the mature form of the Amb a 1.01 isoform (mature protein 26–396 aa, NCBI accession number AAA32665) was designed for insect cell *Spodoptera frugiperda* (*Sf9*) expression. The construct contained an *N*-terminal hexahistidine tag and a sequence (Glu-Asn-Leu-Tyr-Phe-Gln-Gly) for the His-Tag removal protease.

The construct was inserted into a pTM1 vector between the BamHI/SmaI restriction site (ATG:biosynthetics, Merzhausen, Germany) and further introduced into *Escherichia coli* DH10 competent cells (Invitrogen, Thermo Fisher Scientific, Waltham, MA, USA) through a heat-shock transformation, according to the manufacturer’s protocol. The transformed *E. coli* cells were further cultivated on LB agar media containing kanamycin (50 µg/mL), tetracycline (10 µg/mL), gentamicin (7 µg/mL), isopropyl β-D-1-thiogalactopyranoside (IPTG) (40 µg/mL) and X-Gal (100 µg/mL). After a blue–white colony screening, several white colonies were transferred onto new plates and subsequently tested for bacmid insertion by PCR as follows: cells from each colony were resuspended in 10 µL ultrapure water, mixed with 25 µL GoTaq G2 Hot Start Green Mastermix (Promega, Madison, WI, USA), 2.5 µL of M13 Forward primer (5′-CCCAGTCACGACGTTGTAAAACG-3′) and 2.5 µL of M13 Reverse primer (5′-AGCGGATAACAATTTCACACAGG-3′). 

Colonies containing the gene of interest were cultivated overnight in LB medium supplemented with the above-mentioned antibiotics and 40 µg/mL IPTG (Carl Roth, Karlsruhe, Germany) at 37 °C. On the following day, the bacmids were isolated using a Midiprep Kit (Promega, Madison, WI, USA) following the manufacturer’s guidelines. The purified bacmid DNA was subsequently used for the transfection of *Sf9* insect cells (Gibco, Thermo Fisher Scientific, Inc., Waltham, MA, USA) with FuGENE HD transfection reagent (Promega, Madison, WI, USA). The insect cells were cultivated at 27 °C in Sf-900 medium supplemented with 2.5% FBS, 10 µg/mL gentamicin and 250 ng/mL amphotericin B, all from Gibco (Thermo Fisher Scientific, Waltham, MA, USA). After transfection, three steps of baculovirus amplification followed. 

The protein expression was performed for 72 h, the cells were centrifugated and the supernatant (cell medium) was dialyzed overnight against buffer (50 mM NaH_2_PO_4_, 300 mM NaCl, 10 mM imidazole, pH 8). 

### 4.3. Isolation and Purification of Recombinant Amb a 1.01 

The recombinant Amb a 1.01 was purified from the cell culture medium using affinity chromatography with Ni-NTA agarose (Qiagen, Hilden, Germany) according to the manufacturer’s protocol. The elutions with the highest amount of protein were polled and dialyzed against the lysis buffer. The *N*-terminal His-Tag was cleaved by incubating the recombinant protein with AcTEV™ Protease (Invitrogen, Thermo Fisher Scientific, Inc., Waltham, MA, USA) for 6 h at 30 °C. The AcTEV™ Protease and the detached His-Tag were removed from the solution with Ni-NTA agarose. A protease inhibitor (Sigma Aldrich, St. Louis, MO, USA) was added to the protein solution. The protein was dialyzed against 20 mM NaH_2_PO_4_, pH 8, the final buffer. 

### 4.4. Natural Amb a 1.01 Isolation and Ragweed Pollen Extract Preparation 

Natural Amb a 1.01 (nAmb a 1.01) was purified from ragweed pollen extracts by standard chromatography, as described in [21]. The identity, purity, and isoform composition of the nAmb a 1.01 were confirmed by mass spectrometry and it was found to consist of 94% isoform 01, 4% isoform 04, and 1% isoform 02, as described in [21].

The aqueous ragweed pollen extract was obtained from 2 g of ragweed pollen (Allergon AB, Engelholm, Sweden), shaken in 20 mL sterile Dulbecco’s phosphate-buffered saline (DPBS) (Gibco, Thermo Fisher Scientific, Inc., Waltham, MA, USA), pH 7.4, for 4 h at room temperature. Centrifugation at 20,000× *g* for 30 min at 4 °C was employed to eliminate any insoluble material. Subsequently, the pollen extract underwent dialysis against DPBS using a Spectra/Por dialysis membrane with a 3.5 kDa cut-off (Spectrum Labs, Repligen, Waltham, MA, USA) [15,65]. The allergen extract was then stored at –20 °C until use.

### 4.5. Physicochemical Characterization of the Recombinant Amb a 1.01

The concentrations of the natural Amb a 1.01, recombinant Amb a 1.01 and ragweed pollen extract were determined by BCA assay (Pierce, Thermo Fisher Scientific, Inc., Waltham, MA, USA).

For the evaluation of the size and purity aliquots of the expressed protein, 1 μg of rAmb a 1.01 and nAmb a 1 were separated under reducing (sample buffer with ß-mercaptoethanol) and non-reducing (sample buffer without ß-mercaptoethanol) conditions on a 14% SDS-PAGE, followed by Coomassie Brilliant Blue staining. A protein molecular weight marker (PageRuler Plus Prestained Protein Ladder, Thermo Fisher Scientific, Inc., Waltham, MA, USA) was used as a standard for assessing the size of the expressed allergen. The mass of the natural and recombinant Amb a 1.01 was determined through matrix-assisted laser desorption/ionization-time of flight (MALDI-TOF), as described in [66].

An *N*-deglycosylation assay was performed for rAmb a 1.01 using a PNGase A kit according to the manufacturer’s instructions (New England Biolabs, Ipswich, MA, USA). The PNGase A-treated recombinant protein was visualized on SDS-PAGE in comparison to nAmb a 1.01.

The secondary structures of the rAmb a 1.01 and nAmb a 1.01 were analyzed by circular dichroism (CD) spectroscopy using a J-810 spectropolarimeter (Jasco, Easten, MD, USA). The CD spectra of the proteins were measured at concentrations of 0.1 mg/mL in a 0.2 cm quartz cuvette. The far UV spectra were recorded in the range between 190 and 260 nm at room temperature, as described in [67]. The results were expressed as the mean residue ellipticity (θ) at a different wavelength. Based on the CD spectra data, the secondary structures of the two proteins were calculated using the secondary structure estimation program CDSSTR on the DichroWeb server [66,68]. The three-dimensional structural model of Amb a 1.01 was generated by the SWISS-MODEL online tool (https://swissmodel.expasy.org/, accessed on 10 January 2024) [43,69,70].

### 4.6. IgE Reactivity Evaluation of the Recombinant Amb a 1.01 

The IgE reactivity of rAmb a 1.01 was evaluated in comparison to nAmb a 1.01 using both the ELISA and immunoblot methods.

#### 4.6.1. IgE Reactivity by ELISA

Natural Amb a 1.01 and rAmb a 1.01 (5 µg/mL) were each coated overnight at 4 °C on 96-well flat-bottom plates (Maxisorp Nunc, Thermo Fisher Scientific, Inc., Waltham, MA, USA). The plates were washed twice with phosphate-buffered saline (PBS) + 0.05% Tween (PBST) and blocked for 2.5 h with PBST + 3% bovine serum albumin (BSA) at room temperature. The serum samples from 100 ragweed allergic patients, and 5 non-allergic individuals as negative controls, were diluted 1:5 in PBST + 0.5% BSA, added in duplicates on the plates and incubated overnight at 4 °C.

After washing five times with PBST, the bound IgE antibodies were detected with a 1:2500 diluted anti-human IgE horseradish peroxidase (HRP)-linked polyclonal antibody from goat (SeraCare, Milford, MA, USA) by incubating for 45 min at 37 °C and 45 min at 4 °C. After five times washing with PBST, antibody detection was performed by adding 100 µL/well detection substrate 2,2′-Azino-bis(3-ethylbenzothiazoline-6-sulfonic acid) diammonium salt (ABTS) (Sigma Aldrich, St. Louis, MO, USA) in a 60 mM citric acid, 77 mM Na_2_HPO_4_ 2H_2_O solution and 3 mM H_2_O_2_. The absorbance was measured at 405 nm with a reference at 490 nm on a microplate reader (Tecan Infinite M200 Pro, Grödig, Austria). All the ELISA determinations were carried out as duplicates with a deviation of less than 10% of the values and the results are shown as the average of the OD values measured after 10 minutes of reaction. Values greater than the mean value of the 5 negative controls + 3 × standard deviations of the 5 negative controls were considered positive. 

#### 4.6.2. IgE Reactivity by Immunoblotting

Sera from 12 ragweed allergic patients (198, 199, 204, 207, 213, 226, 232, 241, 243, 245, 248, 255) with high (OD > 1.50), medium (OD between 0.50 and 1.50) and weak (OD < 0.50) IgE reactivity, according to the ELISA results, were selected to be tested via immunoblot. Serum from a non-allergic individual and buffer were used as negative controls.

SDS-PAGE (14% SDS polyacrylamide gel) was run with nAmb a 1.01 and rAmb a 1.01 (1 µg) under reducing (R) and non-reducing (NR) conditions. The proteins were blotted onto 0.2 μm nitrocellulose membrane (Amersham Protran, GE Healthcare Life Science, Freiburg, Germany) and cut into 3 mm strips, which were then saturated with buffer A (50 mM Na_2_HPO_4_, 0.6 mM NaH_2_PO_4_, pH 7.5, 0.5% *v/v* Tween-20, 0.5% *w/v* BSA, 0.05% *w/v* NaN_3_). Sera from the Amb a 1.01-positive patients and from the negative control were diluted 1:10 with buffer A and incubated with the strips overnight at 4 °C. After washing with buffer A, the bound IgE was detected using mouse anti-human IgE labelled with AKP (clone G7–26, BD Biosciences, Pharmingen, San Jose, CA, USA) diluted 1:1000 in buffer A. The strips were developed in detection buffer (1.65 mg NBT and 1.65 mg BCIP in 10 mL AP buffer containing 100 mM Tris, 5 mM MgCl_2_ and 100 mM NaCl, pH 9.5).

### 4.7. Allergenic Activity Assessment of the Recombinant Amb a 1.01 Using RBL Mediator Release Assay 

Rat basophil leukemia cells (RS-ATL8) [71] transfected with human FcεRI, kindly provided by Prof. Ryosuke Nakamura, were loaded with serum samples from 8 (199, 204, 207, 213, 226, 241, 248, 255) ragweed allergic patients who were positive to Amb a 1.01 according to the ELISA analysis. The serum samples were diluted 1:10 in MEM medium (Gibco, Thermo Fisher Scientific, Inc., Waltham, MA, USA) supplemented with 10% FBS (Gibco, Thermo Fisher Scientific, Inc., Waltham, MA, USA), penicillin–streptomycin 100 U/mL (Gibco, Thermo Fisher Scientific, Inc., Waltham, MA, USA), 0.2 mM L-Glutamine (Gibco, Thermo Fisher Scientific, Inc., Waltham, MA, USA), geneticin 0.2 mg/mL (Gibco, Thermo Fisher Scientific, Inc., Waltham, MA, USA), and 0.2 mg/mL hygromycin B (Gibco, Thermo Fisher Scientific, Inc., Waltham, MA, USA) and incubated with the cells overnight at 37 °C, 5% CO_2_. The cells were stimulated with serial dilutions of nAmb a 1.01 and rAmb a 1.01 (0.01 ng/mL–1 µg/mL). Buffer without allergens was used as a negative control. For 100% release, the cells were lysed with 10% Triton-X. The release of β-hexosaminidase was measured with a Varioskan LUX reader (Thermo Fisher Scientific, Inc., Waltham, MA, USA) and the results are shown as the percentage of total β-hexosaminidase release [61].

### 4.8. ImmunoCAP IgE Inhibition Experiments

For determining the presence of Amb a 1.01 in the commercially available Amb a 1 tests, 25 µL of serum from 12 Amb a 1.01 allergic patients (198, 199, 204, 207, 213, 226, 232, 241, 243, 245, 248, 255) and from 1 non-allergic individual were preincubated overnight at 4 °C with 75 µL of rAmb a 1.01 (100 µg/mL), ragweed extract (1000 µg/mL) and DPBS buffer control. After pre-incubation, the sera was tested for IgE binding to commercially available nAmb a 1 ImmunoCAPs, w230 (Thermo Fisher Scientific, Inc., Waltham, MA, USA) according to the manufacturer’s guidelines. The IgE reactivity was measured using a Phadia 250 analyzer (Thermo Fisher Scientific, Inc., Waltham, MA, USA). The IgE values of the sera pre-incubated with DPBS were considered the baseline and were also used for calculating the inhibition percentages. 

The IgE inhibition was calculated as (1–rAmb a 1.01/Buffer) × 100 in the case of pre-incubation with rAmb a 1.01, and (1–ragweed extract/Buffer) × 100 in the case of pre-incubation with ragweed extract. 

### 4.9. Statistical Analysis

The OD values measured in the ELISA were tested for normal distribution using the Shapiro–Wilk test. The rAmb a 1.01 OD values were compared with nAmb a 1.01 by Sperman’s correlation, where a Spearman’s rho value greater than 0.7 was considered a strong correlation. The data analysis was performed in GraphPad Prism 7.0 (GraphPad Software, Inc., La Jolla, CA, USA). The results were considered statistically significant if the *p*-value was below 0.05.

## 5. Conclusions

Our study represents a step forward in the recombinant expression of Amb a 1.01, an isoform of the major ragweed pollen allergen. The recombinant Amb a 1.01 produced in insect cells behaves similarly to the natural isoform and represents a useful tool for further diagnostic and research applications. Furthermore, the availability of rAmb a 1.01 is a major step forward in overcoming the challenges associated with the variability of natural allergen extracts. 

## Data Availability

The original contributions presented in the study are included in the article and Supplementary Material.

## Supplementary Materials

The following supporting information can be downloaded at https://www.mdpi.com/article/10.3390/ijms25105175/s1.
