# Supplementary material for: Insect Cell-Expressed Major Ragweed Allergen Amb a 1.01 Exhibits Similar Allergenic Properties to Its Natural Counterpart from Common Ragweed Pollen
Source: Int J Mol Sci. 2024 May 9;25(10):5175. doi: 10.3390/ijms25105175 (PMC11121294; doi:10.3390/ijms25105175)
Supplement: Supplementary file 1 [file ijms-25-05175-s001.zip › ijms-2940023-supplementary.pdf]

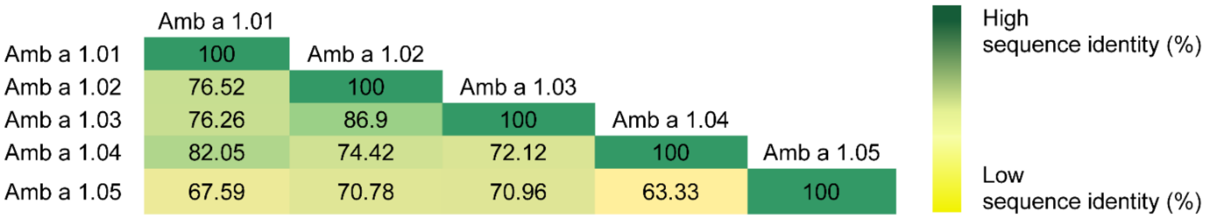

(**a**)

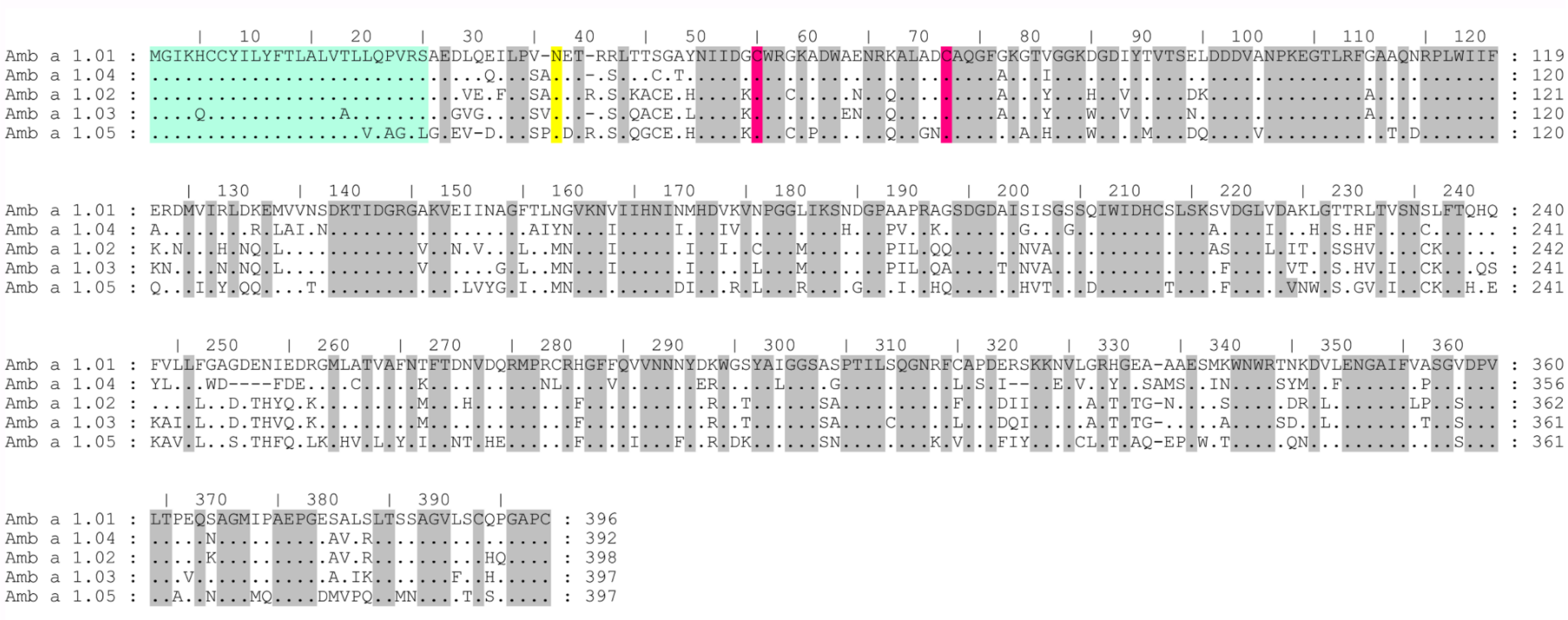

(**b**)

Figure S1. Sequence similarity of Amb a 1.01 with other Amb a 1 isoforms. (**a**) Sequence identity matrix of Amb a 1 isoforms. The identity percentage was determined by Clustal Omega. (**b**) Sequence alignment of Amb a 1.01 with other Amb a 1 isoforms. The

isoform sequences were ordered according to the percentage of homology with Amb a 1.01 isoform. The identical sequences were highlighted in grey, the N-glycosylation site was highlighted in yellow, the cysteine residues reported to form-the disulphide bonds in Amb a 1.01 were highlighted in pink and the signal peptide was highlighted in blue. The sequence alignment was performed in GeneDoc.

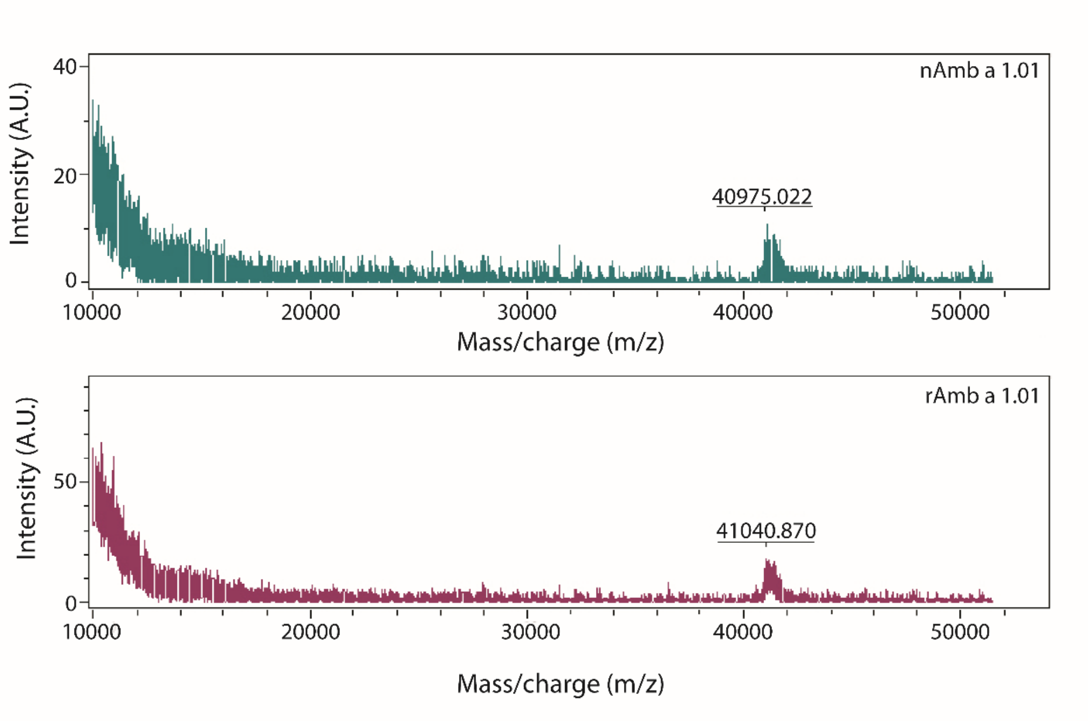

Figure S2. MALDI-TOF analysis of nAmb a 1.01 and rAmb a 1.01. The mass/charge ratio is indicated on the x-axis, and intensity is shown as absorption units (A.U.) of the most intensive signals obtained in the mass range (y-axis)

Table S1. IgE reactivity of rAmb a 1.01 and nAmb a 1.01 in ELISA and other sensitization of the patients included in the study.

Patient number	rAmb a 1.01* (cut off = 0.09)	nAmb a 1.01* (cut off = 0.06)	Other sensitization than ragweed pollen
156	1.177	1.175	-
157	0.752	0.663	Hazel, Birch, Wheat, Grass pollen mix, Peanuts, Hazelnuts
158	0.109	0.127	Grass pollen mix, HDM, Cat, *Blatella*
159	0.292	0.359	Grass pollen mix, HDM, Cat, Dog
160	0.905	0.666	-
161	0.208	0.293	-
162	0.386	0.404	Hazel, Birch, Beech, Wheat, Grass pollen mix, Mugwort
163	0.511	0.562	Hazel, Birch
164	0.908	0.928	Wheat, Grass pollen mix, Mugwort, HDM, Cat, Dog
165	0.347	0.411	-
166	0.445	0.576	-
167	0.292	0.358	Birch, Lawn grass
168	0.641	0.950	-
169	0.643	0.849	Wheat, HDM, *Aspergillus*, *Alternaria*
170	1.098	1.205	-
171	0.061	0.047	-

**172**	**0.510**	**0.491**	Wheat, Timothy grass, Barley, Orchard grass
**173**	**0.132**	**0.108**	-
**174**	**1.407**	**1.487**	Birch, Lawn grass, Timothy grass, Barley, Orchard grass, HDM
**175**	**0.546**	**0.514**	-
**176**	**0.903**	**0.906**	Hazel, Wheat, Barley, Mugwort, HDM
**177**	**0.499**	**0.425**	HDM
**178**	**0.169**	**0.159**	Birch, Lawn grass, Barley, Orchard grass
**179**	**0.430**	**0.485**	Birch, Wheat, Grass pollen mix, Mugwort
**180**	**0.583**	**0.813**	-
**181**	**0.250**	**0.380**	Hazel, Lawn grass
**182**	**0.603**	**0.864**	Mugwort, HDM, *Alternaria, Candida*
**183**	**0.872**	**1.021**	Timothy grass, Dog
**184**	**0.426**	**0.477**	-
**185**	**0.345**	**0.454**	-
**186**	**0.211**	**0.294**	-
**187**	**0.241**	**0.276**	Wheat, Grass pollen mix, Cat
**188**	**0.551**	**0.462**	Beech
**189**	**0.141**	**0.115**	Birch, Wheat, HDM
**190**	**1.354**	**1.865**	Dactylis glomerata, Barley, Mugwort, Blatella, *Candida*
**191**	**0.610**	**0.607**	HDM
**192**	**0.245**	**0.250**	HDM
**193**	**0.466**	**0.479**	Mugwort, HDM
**194**	**0.800**	**0.927**	HDM
**195**	**1.971**	**2.208**	-
**196**	**0.338**	**0.555**	Timothy grass, Lawn grass, Barley, HDM
**197**	**0.103**	**0.144**	HDM
**198**	**1.327**	**1.217**	-
**199**	**2.581**	**2.527**	Hazel, Wheat, Timothy grass, Lawn grass, Dactylis glomerata, Barley, Mugwort
**200**	**0.180**	**0.175**	HDM
**201**	**0.494**	**0.495**	Grass pollen mix, Barley,
**202**	**0.650**	**0.629**	Wheat, Lown grass, Mugwort
**203**	**0.836**	**1.032**	Mugwort
**204**	**1.646**	**1.500**	Mugwort
**205**	**0.818**	**0.615**	Grass pollen mix, Lawn grass, Timothy grass, Dactylis glomerata, Mugwort
**206**	**0.414**	**0.198**	-
**207**	**1.167**	**1.293**	Mugwort, HDM
**208**	**0.326**	**0.381**	Mugwort
**209**	**0.344**	**0.336**	HDM

**210**	**1.331**	**1.445**	Hazel, Birch, Wheat, Grass pollen mix, Rye, Mugwort, HDM
**211**	**0.494**	**0.711**	Grass pollen mix
**212**	**0.123**	**0.145**	Hazel, Birch, Wheat, Grass pollen mix, Lawn grass, Timothy grass, Barley, Cat
**213**	**2.811**	**2.643**	-
**214**	**0.554**	**0.607**	-
**215**	**0.486**	**0.469**	Grass pollen mix, HDM
**216**	**0.560**	**0.188**	-
**217**	**0.930**	**1.237**	HDM
**218**	**1.088**	**1.261**	-
**219**	**2.419**	**2.598**	Wheat, Lown grass, Timothy grass, Barley, Dactylis glomerata, Mugwort
**220**	**2.394**	**2.305**	HDM
**221**	**1.197**	**1.365**	-
**222**	**1.250**	**1.995**	Hazel, Birch, Wheat, Grass pollen mix, Lawn grass, Timothy grass, Barley, Dactylis glomerata, Mugwort, HDM
**223**	**1.112**	**1.321**	-
**224**	**0.611**	**1.056**	-
**225**	**0.144**	**0.200**	Hazel, Birch
**226**	**0.569**	**0.495**	-
**227**	**0.661**	**0.724**	Lawn grass
**228**	**1.161**	**1.232**	Wheat, Grass pollen mix, Lawn grass, Timothy grass, Barley, Dactylis glomerata, Blatella
**229**	**1.230**	**1.285**	Wheat, Grass pollen mix, Lawn grass, Timothy grass, Barley, Dactylis glomerata,
**230**	**0.144**	**0.180**	-
**231**	**0.738**	**1.049**	-
**232**	**0.612**	**0.564**	-
**233**	**1.041**	**1.102**	-
**234**	**0.374**	**0.469**	Barley, Mugwort
**235**	**0.947**	**1.063**	Hazel, Birch, Barley, HDM
**236**	**2.592**	**2.706**	Grass pollen mix, Barley, HDM
**237**	**1.235**	**1.573**	-
**238**	**1.885**	**2.293**	Hazel, Birch, Grass pollen mix, HDM, Cat
**239**	**1.281**	**1.294**	Timothy grass, Dactylis glomerata, Mugwort
**240**	**1.413**	**1.092**	-
**241**	**0.851**	**0.882**	-
**242**	**0.907**	**0.936**	-
**243**	**0.823**	**0.950**	Dog
**244**	**0.222**	**0.234**	-
**245**	**0.119**	**0.094**	-

	**246**	**0.996**	**1.335**	Hazel, Birch, Wheat, Grass pollen mix, Barley
	**247**	**1.719**	**1.993**	Wheat, Lawn grass, Barley
	**248**	**0.347**	**0.489**	Grass pollen mix, Barley
	**249**	**1.436**	**1.411**	Wheat, Grass pollen mix, Barley, Mugwort, Cat
	**250**	**1.578**	**1.610**	Hazel, Birch, Grass pollen mix, HDM, Alternaria
	**251**	**0.601**	**0.877**	-
	**252**	**1.009**	**1.367**	-
	**253**	**1.079**	**1.241**	HDM, Alternaria
	**254**	**0.333**	**0.518**	HDM
	**255**	**0.367**	**0.368**	Hazel, Birch, Grass pollen mix, Barley
**Total**	**100**	**99**	**99**	

**Sensitization profile**	
**Monosensitized**	**37%**
**Polysensitized**	**63%**
**Mugwort pollen**	**20%**
**Tree pollen**	**21%**
**Grass pollen**	**42%**
**HDM**	**29%**
**Fungi**	**5%**
**Cat**	**7%**
**Dog**	**4%**
Blatella	**3%**

ELISA positive values were marked with the bold and red color font *mean OD values of the duplicates. HDM - house dust mite; Grass pollen mix: Orchard grass, Lawn grass, Red fescue, Rye, Timothy grass, Meadow soft grass

Table S2. Mediator release assay results after stimulation with nAmb a 1.01 and rAmb a 1.01

**Allergen concentration**	**Patient 199**		**Patient 204**		**Patient 207**		**Patient 213**		**Patient 226**		**Patient 241**		**Patient 248**		**Patient 255**	
	**nAmb a 1.01**	**rAmb a 1.01**	**nAmb a 1.01**	**rAmb a 1.01**	**nAmb a 1.01**	**rAmb a 1.01**	**nAmb a 1.01**	**rAmb a 1.01**	**nAmb a 1.01**	**rAmb a 1.01**	**nAmb a 1.01**	**rAmb a 1.01**	**nAmb a 1.01**	**rAmb a 1.01**	**nAmb a 1.01**	**rAmb a 1.01**
**0**	6.03	6.03	2.94	2.94	4.67	4.67	2.74	2.74	5.1	5.1	3.87	3.87	3.71	3.71	4.84	4.84
**0.01**	12.05	6.91	5.6	4.86	6.31	4.96	9.39	6.34	7.42	5.37	4.02	3.23	3.67	3.42	4.72	3.28
**0.1**	27.19	8.55	15.24	6.61	9.62	5.43	40.33	18.65	5.83	4.93	5.87	3.36	5.61	3.79	4.38	4.68
**1**	44.31	29.55	45.34	20.41	31.25	10.26	59.95	48	12.31	5.55	17.16	4.9	3.93	4.53	4	3.32
**10**	59.57	57.99	63.63	64.28	52.3	41.17	60.47	69.39	36.42	20.17	43.87	25.9	13.56	8.06	14.02	6.71
**100**	58.23	69.44	62.28	80.46	51.09	67.32	55.6	72.1	40.19	41.85	58.88	60.55	21.72	22.26	20.53	21.59
**1000**	58.69	75.25	63.61	86.14	60.93	73.45	50.43	71.45	44.99	55.32	62.49	71.46	17.82	26.74	18.66	24.11

The green color marks an RBL degranulation above 20%.

Table S3. ImmunoCAP IgE inhibition results after sera incubation with buffer, rAmb a 1.01 and ragweed pollen extract

Patient	**Buffer** (IgE level - kUA/L)	**rAmb a 1.01** (IgE level - kUA/L)	**Ragweed pollen extract** (IgE level - kUA/L)	**rAmb a 1.01** (Inhibition %)	**Ragweed pollen extract** (Inhibition %)
**198**	11.093	0.657	0.934	94.07	91.58
**199**	52.807	7.43	8.047	85.93	84.76
**204**	17.708	2.052	2.292	88.41	87.05
**207**	20.14	1.191	1.757	94.08	91.27
**213**	102.769	24.742	25.447	75.92	75.23
**226**	9.239	1.016	1.509	89.00	83.66
**232**	5.241	0.881	0.885	83.19	83.11
**241**	9.969	0.865	0.726	91.32	92.71
**243**	10.541	0.817	1.326	92.24	87.42
**245**	0.383	0.006	0.035	98.43	90.86
**248**	4.335	0.256	0.656	94.09	84.86
**255**	4.466	0.397	0.458	91.11	89.74
**NC**	0	0.004	0	-	-
			**Mean inhibition (%)**	**89.82**	**86.86**

NC – negative control
